# Comprehensive molecular expression profiling of SARS-CoV-associated factors in the endometrium across the menstrual cycle and elevated susceptibility in women with recurrent pregnancy loss

**DOI:** 10.3389/fgene.2023.1246725

**Published:** 2023-10-03

**Authors:** Ruofan Qi, Rui Guan, Shengyun Cai, Mingjuan Xu, Wen-jui Yang, Chi Chiu Wang

**Affiliations:** ^1^ Department of Obstetrics and Gynaecology, The Chinese University of Hong Kong, Shatin, China; ^2^ Department of Obstetrics and Gynecology, Changhai Hospital, Second Military Medical University, Shanghai, China; ^3^ Department of Infertility and Reproductive Medicine, Taiwan IVF Group Center, Hsinchu, Taiwan; ^4^ Department of Fertility and Reproductive Medicine, Ton-Yen General Hospital, Hsinchu, Taiwan; ^5^ Li Ka Shing Institute of Health Sciences, The Chinese University of Hong Kong, Hong Kong, China; ^6^ School of Biomedical Sciences, The Chinese University of Hong Kong, Hong Kong, China; ^7^ Chinese University of Hong Kong-Sichuan University Joint Laboratory in Reproductive Medicine, The Chinese University of Hong Kong, Hong Kong, China

**Keywords:** COVID-19, endometrial gene expression, peri-implantation period, recurrent implantation failure, recurrent pregnancy loss COVID-19, recurrent pregnancy loss

## Abstract

**Objective:** To evaluate the dynamic expression profiling alterations of SARS-CoV-2-associated molecules within the fertile human endometrium throughout the menstrual cycle. Furthermore, to explore the inherent vulnerability of the endometrium to SARS-CoV-2 infection among women experiencing recurrent pregnancy failure, including both recurrent implantation failures (RIF) and recurrent pregnancy losses (RPL).

**Method:** The present study employed multiple datasets to investigate the expression patterns of SARS-CoV-2-associated genes. Firstly, a single-cell RNA-sequencing dataset comprising endometrial samples from 19 healthy women across the menstrual cycle was utilized. Additionally, two microarray datasets encompassing 24 women with RIF, and 24 women with RPL during the peri-implantation phase were included. To complement these analyses, immunohistochemical (IHC) staining was performed on endometrial samples collected from 30 women with RIF, 30 women with RPL, and 20 fertile controls recruited specifically during the implantation period.

**Results:** The investigation revealed a moderate expression percentage of CTSL (22%), TMPRSS4 (15%), FURIN (16%) and MX1 (9%) in endometrium. Conversely, the expression percentages of ACE2 (1%) and TMPRSS2 (4%) were relatively low. Notably, the expression of BSG exhibited an increment towards the window of implantation, reaching its peak during the middle secretary phase. Furthermore, a significant reduction (*p* < 0.05) in TMPRSS2 expression was observed in the RIF group compared to the control group. While the expression of BSG was significantly increased (*p* < 0.05) in the RPL group, findings that were corroborated by the IHC staining results.

**Conclusion:** The findings of this study indicate a noteworthy upregulation of BSG expression in the endometrium of women with RPL. These results suggest an augmented susceptibility of endometrium to SARS-CoV-2 infection, potentially contributing to unfavorable pregnancy outcomes.

## Introduction

The global COVID-19 pandemic caused by severe acute respiratory syndrome coronavirus 2 (SAR-CoV-2) has significantly impacted public health and lifestyles worldwide, with COVID-19 exerting a great impact on many people’s health and lifestyle worldwide. Since its emergence in 2019, SARS CoV-2 has presented a substantial challenge for healthcare professionals due to its high transmissibility and associated sequelae. Consequently, there is a growing need to understand the impact of this virus on women’s reproductive health. Notably, pregnant women have been identified as a particularly susceptible population to respiratory pathogens, with and increased risk of severe pneumonia owing to immune and cardiopulmonary system changes (Liu et al., 2020). As such, pregnant women face heightened vulnerability during the COVID-19 pandemic, necessitating a comprehensive assessment of the risks posed by exposure to COVID-19 and its effects on pregnancy. Insufficient reliable data on the effects of COVID-19 on embryo implantation may raise concerns among women with fertility requirements, particularly those with a history of recurrent pregnancy failures. Therefore, the current study aimed to investigate SARS-CoV-2 related molecules within the human endometrium of women experiencing recurrent pregnancy failures, including both recurrent implantation failures (RIF) and recurrent pregnancy losses (RPL).

A set of crucial genes involved in the viral entry and processing mechanisms utilized by SARS-CoV-2 to infect host cells has been identified. The virus primarily enters host cells through two main receptors: angiotensin-converting enzyme 2 (ACE2), a critical component of renin-angiotensin system (RAS) (Hoffmann et al., 2020); and basigin (BSG), also known as a cluster of differentiation 147 (CD147) or extracellular matrix metalloproteinase inducer (MMPRIN), which is a putative receptor (Wang et al., 2020a; Ulrich and Pillat, 2020). For viral entry through ACE2, the transmembrane spike glycoprotein (S protein) of SARS-CoV-2 is primed by transmembrane protease serine 2 (TMPRSS2) and transmembrane protease serine 4 (TMPRSS4). This priming enables the fusion of viral and cellular membranes, allowing for viral entry and replication in target cells (Glowacka et al., 2011). Additionally, lysosomal cysteine proteases, namely, cathepsin B (CTSB) and cathepsin L (CTSL), are believed to facilitate the intracellular processing of the SARS-CoV-2 spike protein, serving as alternative mechanisms in the absence of TMPRSS2 (Hoffmann et al., 2020). FURIN, also known as paired basic amino acid cleaving enzyme (PACE), plays a critical role in the proteolytic activation of SARS-CoV-2 in human epithelial cells. It possesses a unique cleavage site specific to SARS-CoV-2, absent in other beta-coronavirus subgenus, which contributes to the virus’s relatively high infectivity (Bestle et al., 2020; Coutard et al., 2020). BSG has been reported to significantly participate as an alternative virus receptor for ACE2-defiant cells in SARS-CoV-2 infection (Wang et al., 2020b). Moreover, immune cells such as T-cell, B-cell, macrophages, and natural killer cells may disseminate the virus via BSG in infected epithelial cells, resulting local and systemic virus dissemination throughout the body (Peng et al., 2020). The myxo virus resistance protein 1 (Mx1), a dynamin-like guanosine triphosphatase, impedes early stages of the viral replication cycle by binding to virus nucleoproteins. It is widely known for its antiviral activity against RNA and DNA viruses (Haller et al., 2015). It is plausible that the expression of these genes in cells of the female reproductive system following viral infection could impact reproductive function. It remains under investigation whether the virus can infiltrate and infect the endometrium, potentially altering the local environment and subsequently influencing various processes such as embryo implantation and development, leading to implantation failure or miscarriage. Therefore, considering that the ability of SARS-CoV-2 to affect specific tissues or cells is contigent upon its capacity to enter and replicate within them, we assessed expressions of SARS-CoV-2- related molecules in the endometrium during the implantation window among different groups of women in this study.

To elucidate the plausibility of endometrial transmission of SARS-CoV-2, we initiated our investigation by thoroughly analyzing the characteristics and distribution patterns of potential SARS-CoV-2 related molecules within the human endometrium. This analysis was conducted using single-cell RNA-sequencing (scRNA-seq) technology. Subsequently, we examined the expression profiles of SARS-CoV-2-related molecules in women with recurrent reproductive failure, utilizing microarray data derived from endometrial samples. To validate and reinforce the findings obtained through bioinformatic analysis, we performed immunohistochemical staining on endometrial samples obtained from distinct study groups, including women with recurrent implantation failures (RIF), recurrent pregnancy losses (RPL), and fertile controls prospectively recruited for this study.

## Methods

A comprehensive bioinformatics analysis was conducted based on 3 publicly availabe datasets. Firstly, a search was performed on the NCBI Gene Expression Omnibus (GEO) database (https://www.ncbi.nlm.nih.gov/geo/) using the key words “endometrium” and “single cell”, then a separate search using key words “endometrium” and “RIF” was conducted, finally another search was carried out using key words “endometrium” and “RPL”. The screening criteria for dataset selection were as follows: i) the organism must be restricted to ‘*homo sapiens*’; ii) a complete expression profile determination of the endometrium had to be performed; and iii) all datasets needed to contain at least five samples.

Upon careful review, three gene expression profiles were identified and downloaded from the GEO Datasets database. Among them, GSE111976 was based on GPL18573 platform (Illumina NextSeq 500, *Homo sapiens*) and GPL24676 platform (Illumina NovaSeq 6000 *Homo sapiens*). GSE111974 was based on platform GPL17077 (Agilent-039494 SurePrint G3 Human GE v2 8 × 60K Microarray 039381). GSE165004 was based on the GPL16699 platform (Agilent-039494 SurePrint G3 Human GE v2 8 × 60K Microarray 039381).

scRNA-seq analysis was conducted using Dataset GSE111976, which involved endometrial biopsies obtained from 19 healthy ovum donors between 4–27 days after the onset of menstrual bleeding. Inclusion criteria for this dataset comprised women with a normal karyotype and negative serological tests for human immunodeficiency virus, hepatitis B virus, hepatitis C virus and syphilis. Women with recent contraception use, uterine pathology or polycystic ovary syndrome were excluded. In total, 2,148 single cells expressing 56,401 genes were extracted from 19 healthy human endometria across the menstrual cycle were extracted.

For dataset GSE111974, inclusion criteria for RIF (n = 24) encompassed women who experienced failure of pregnancy in three consecutive *in vitro* fertilization (IVF) cycles, with each cycle involving the transfer of good quality embryos. Exclusion criteria consisted of active pelvic infections, undiagnosed vaginal bleeding, uterine anomalies, endometriosis, and karyotype anomalies in either partner.

In the case of dataset GSE165004, the inclusion criteria for RPL (n = 24) entailed women under 35 years with regular menstrual cycle, experiencing at least two consecutive pregnancy losses before 20 weeks of gestation. Additional criteria included normal levels of follicle-stimulating hormone (FSH), luteinizing hormone (LH), estradial (E2), prolactin (PRL), and thyroid stimulating hormone (TSH) on days 2-3 of the menstrual cycle, normal uterine cavity shape and size, and bilateral tubal patency observed on hysterosalpingogram, as well as normal antithrombin III, protein C and protein S activity. The women and their partners were required to have normal karyotypes and sperm parameters. Exclusion criteria encompassed the detection of mutations in Factor V (Leiden) and prothrombin gene analysis, positive results for lupus anticoagulant evaluation, cardiolipin antibody (IgM and IgG), and beta2-glycoprotein antibody (IgM and IgG).

The inclusion criteria for the fertile control group in both the GSE111974 and GSE165004 datasets consisted of women under 35 years with regular menstrual cycles and a history of at least one live birth. Exclusion criteria compassed women with a history of infertility treatment, previous miscarriages associated gynecologic comorbidities or ongoing medication usage. And all the endometrial samples included in the GSE111974 and GSE165004 datasets were taken specifically between days 19 and 21 of the menstrual cycle.

In addition, a pilot cohort study was conducted, recruiting a total of 80 participants from the Prince of Wales Hospital, The Chinese University of Hong Kong in Hong Kong. This cohort included 20 fertile control, 30 women with RM and 30 women with RIF. The recruitment of participants took place at LH+7 days of the menstrual cycle, and endometrial samples were collected for immunohistochemical staining. Fertile controls were defined as women who had at least one live born without any major pregnancy complications. RM was defined as women with a history of three consecutive miscarriages before reaching gestational week 20, while RIF was defined as women who failed to achieve a clinical pregnancy despite the transfer of at least four morphologically good-quality embryos in a minimum of three cycles. Ethical approval for this study was obtained from the Joint Chinese University of Hong Kong New Territories East Cluster Clinical Research Ethics Committee (CREC Ref. No. 2016.127), and all participants provided informed consent prior to their inclusion in the study.

### Preprocessing and visualization of RNA-seq data

Single cell data was primarily analyzed using Seurat R package. The “FindClusters” function was employed to identify and visualize 7 distinct cell clusters through the t-SNE (t-Distributed Stochastic Neighbor Embedding) method. To determine specific markers for each cluster, gene expression within individual clusters was examined. Known markers of various cell types, sourced from published literature and specific databases, were then mapped to the predicted markers in Seurat for the purpose of assigning cell types to the different clusters. Microarray datasets were processed using R version 3.6.2 and RStudio 1.1.456 along with the Seurat and ggplot2 packages.

### Paraffin sections and immunohistochemical staining

Paraffin sections were prepared following the protocal described by Hoffmanet. Briefly, tissue samples were collected, fixed, dehydrated and cleared before being embedded in paraffin. Subsequently, 5 μm sections were obtained from the embedded tissues of fertile controls, RIF and RPL, which were then mounted on the same glass slide for subsequent immunohistochemical staining. The sections underwent deparaffinization, hydration, blocking of endogenous peroxidase activity, and antigen retrieval before being incubated with antibodies against TMPRSS2 (Abcam, ab109131) and BSG (Abcam, ab108308). Color staining was achieved using 3.3-diaminobenzidene tetrahydrochloride (DAB, Dako) substrate solution. After another round of incubation with blocking solution and biotinylated secondary antibody, the sections were counterstained, dehydrated, cleared and cover slipped for long-term storage at room temperature. Imaging of the sections was performed using a Leica scanner (Leica, Wetzlar, Germany) according to standard microscopic examination procedures. Five fields, including luminal epithelium (LE), glandular epithelium (GE) and stroma (ST) of the endometrium, were captured in each section under ×400 magnification. The localization and expression levels of each protein in different compartments of the endometrium were examined, and their expression levels were quantified using inForm 2.4 Software (Perkin‐Elmer/Caliper Life Science, United States). InForm software automatically identifies different tissue types and distinguishes epithelial (glandular and luminal) cells from stroma cells based on user-defined training regions. The immunohistochemical signals’ intensity within each compartment was classified into four levels: 0, 1+, 2+ and 3+, and H-score was generated by multipling the percentages of positively stained cells, which corresponded to the levels of absent (0%), weak (30%), moderate (50%), or strong (70%).

### Statistics

Data were analyzed using GraphPad Prism Software version 6.0. The Shapiro-Wilk test was employed to assess data distribution. Quantitative data were presented as mean ± standard error of mean (SEM). Differences between groups were assessed using independent *t*-test, chi-square test or Mann-Whitney *U* test as appropriate. A probability value below 0.05 (*p* < 0.05) was considered statistically significant.

## Results

### t-SNE mapping of viral infectivity genes in endometrium cells

**FIGURE 1:**
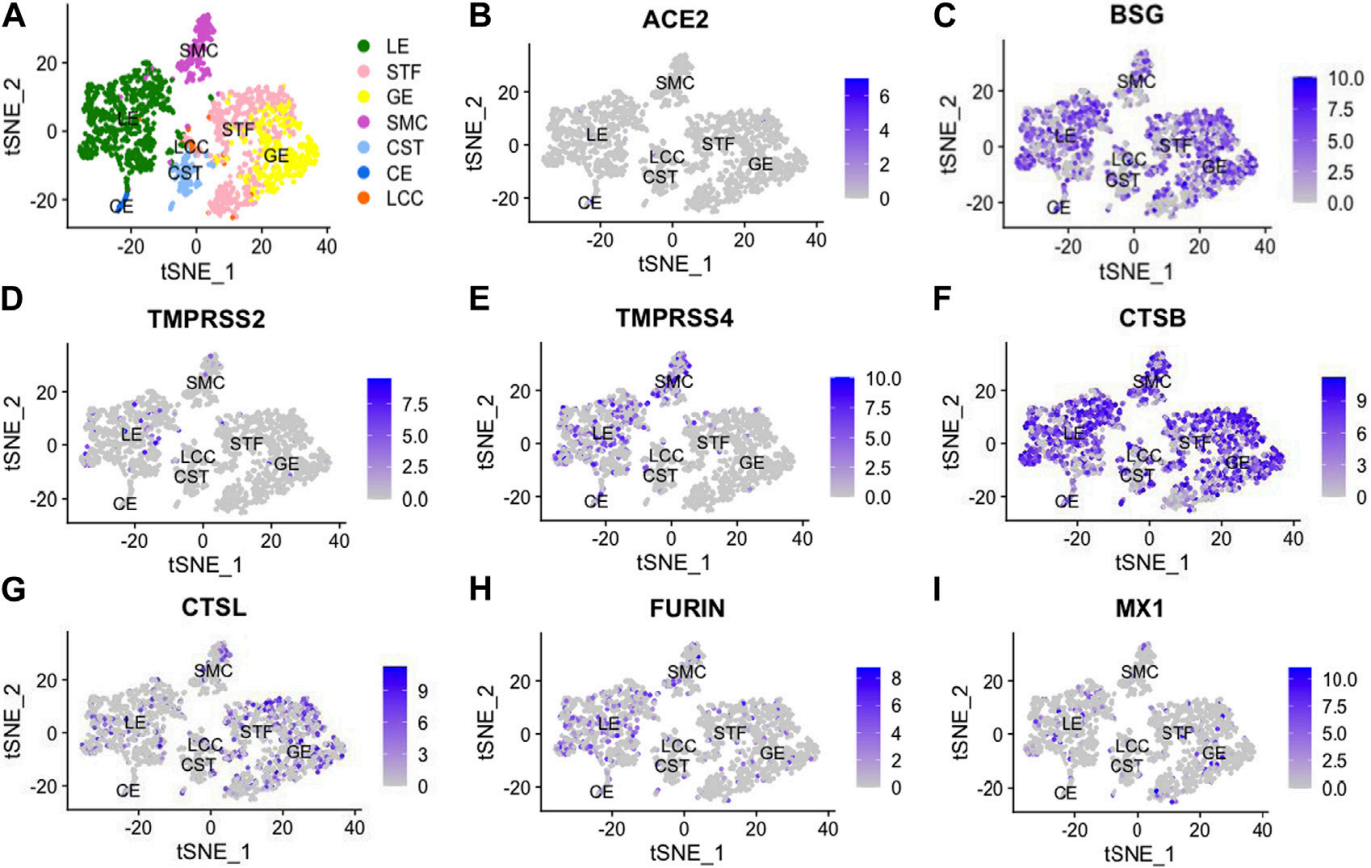
t-Distributed Stochastic Neighbor Embedding (t-SNE) distributions showing cell clusters **(A)** of 2,148 single cells from 19 healthy human endometria across the menstrual cycle. In total, 7 cell types were identified, including unciliated luminal epithelium (LE), stromal fibroblast (STF), unciliated glandular epithelium cells (GE), smooth muscle cells (SMC), cycling stroma cells (CST), ciliated epithelial cells (CE) and lymphocyte cells (LCC). Additionally, we performed dimension reduction (t-SNE) analysis for SARS-CoV-2-related genes, including angiotensin-converting enzyme 2 (ACE2), basigin (BSG), transmembrane protease serine 2 (TMPRSS2), transmembrane protease serine 4 (TMPRSS4), cathepsin B (CTSB), cathepsin L (CTSL), FURIN and myxovirus resistant 1 (MX1), as shown in **(B -I)**.

By analyzing previously published single-cell transcriptomic profiles, we isolated 2,148 individual cells expressing 56,401 genes from 19 healthy human endometrial samples obtained across the menstrual cycle. In total, seven distinct cell types were identified, namely, unciliated luminal epithelium cells (LE), stromal fibroblasts (STF), unciliated glandular epithelium cells (GE), smooth muscle cells (SMC), cycling stroma cells (CST), ciliated epithelium cells (CE) and lymphocyte cells (LCC) (Figure 1).

### Comparison of viral infectivity genes expression in endometrium cells

**FIGURE 2:**
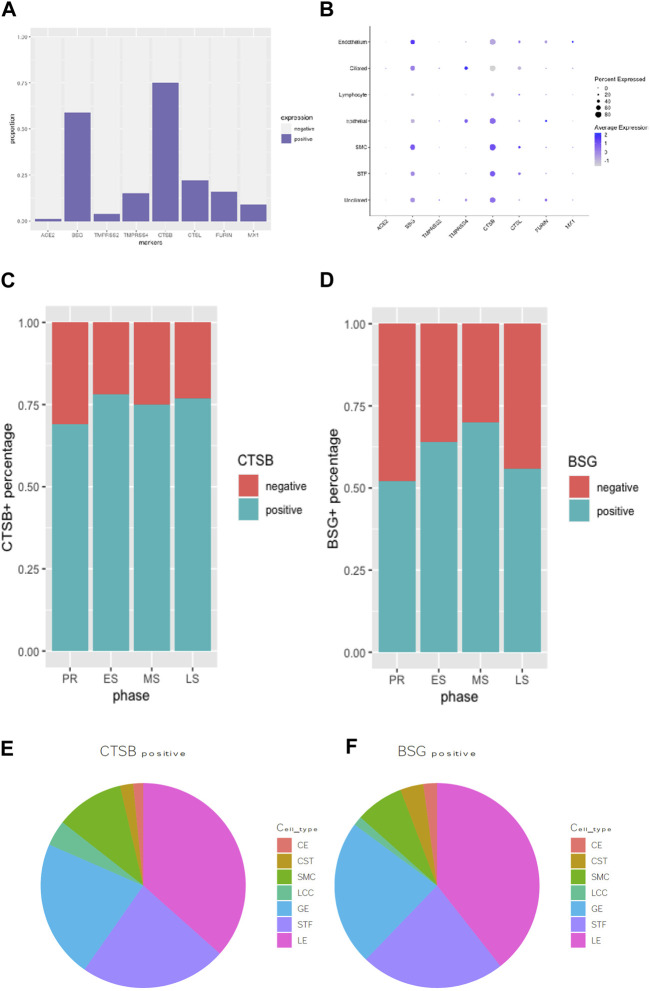
Expression proportion of various SARS-CoV-2-related molecules in endometrial cells were compared using bar plot **(A)**. A bubble plot illustrates the expression levels of various viral infectivity genes in each cell type in endometrium **(B)**. CTSB+ **(C)** and BSG+ **(D)** distribution during the menstrual cycle were displayed in bar graphs. The pie graph showed specifically distributions of the most expressed “CTSB” **(E)** and “BSG” **(F)** genes in different endometrial cell types.

All viral infectivity genes, including ACE2, BSG, TMPRSS2, TMPRSS4, CTSB, CTSL, FURIN and MX1 were expressed in the endometrium. Among them, CTSB (75%) and BSG (59%) showed relatively high expression percentage in endometrium cells. Moderately expressed viral infectivity genes included CTSL (22%), TMPRSS4 (15%), FURIN (16%) and MX1 (9%), whereas ACE2 (1%) and TMPRSS2 (4%) demonstrated lower expression percentages (Figures 2A, B). We further grouped the cells based on menstrual phases and calculated the positive proportions of CTSB (Figure 2C) and BSG (Figure 2D) in each menstrual phase, including the proliferative phase (PR), early secretary phase (ES), middle secretary phase (MS) and late secretary phase (LS). There was elevated expression of CTSB in early secretary phase of the natural menstrual cycle, while BSG expression increased toward the window of implantation and reached its peak during the middle secretary phase. Both CTSB and BSG were predominantly expressed in unciliated epithelium cells, stroma fibroblasts and smooth muscle cells in the endometrium (Figures 2E, F).

### Comparisons of viral infectivity genes in endometrium between fertile controls (Con) and women with recurrent implantation failure (RIF), and fertile controls (Con) and women with recurrent pregnancy loss (RPL).

**FIGURE 3:**
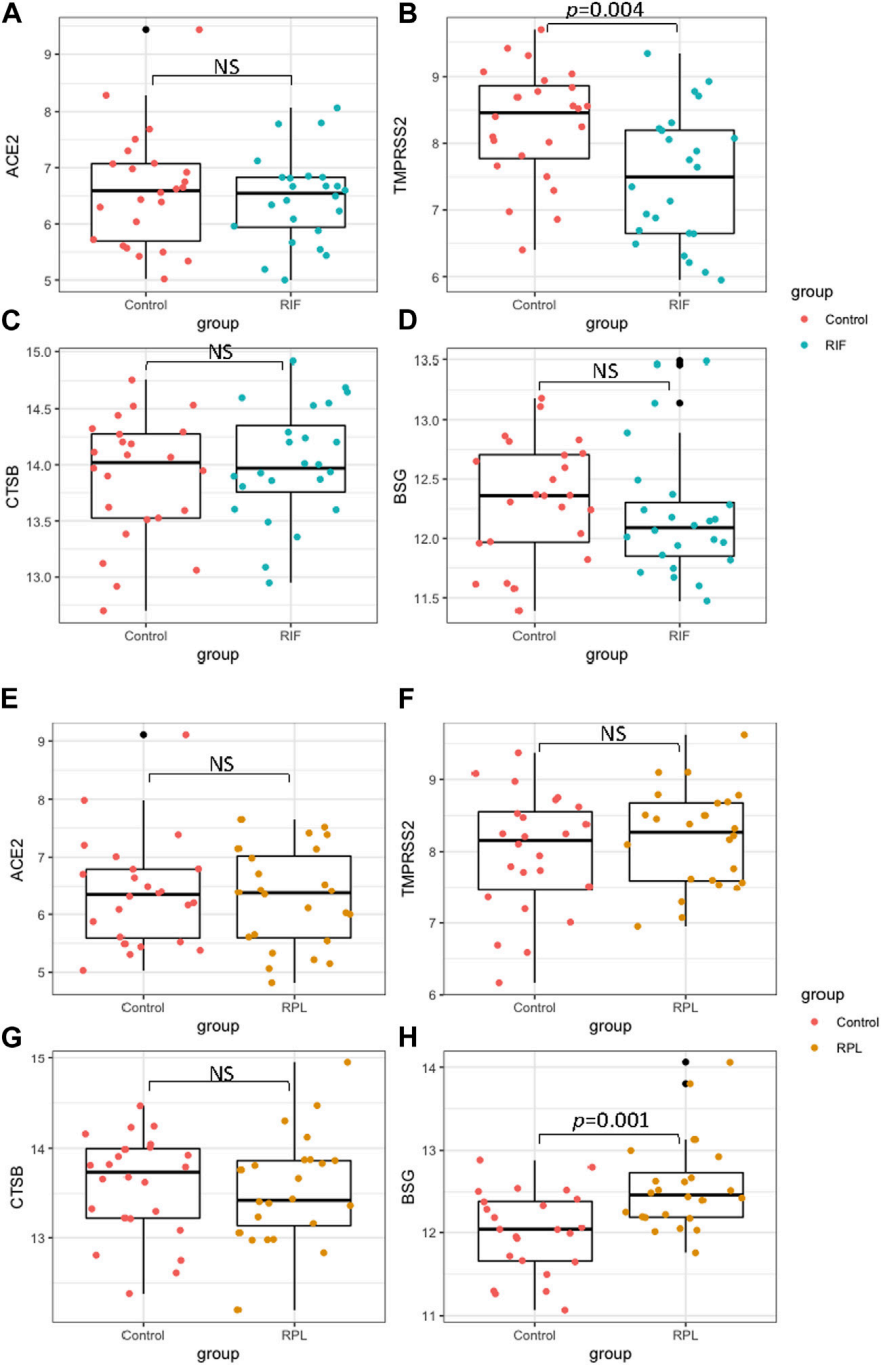
The expression of ACE2, TMPRSS2, CTSB and BSG in mid-secretary phase endometrium were compared between fertile controls with RIF (panels **(A–D)**), as well as with RPL **(E–H)**).

The expression of ACE2, TMPRSS2, CTSB and BSG in the mid-secretary phase of the endometrium was compared between Con and RIF, as well as between Con and RPL. Detailed demographic characteristics of the women who participated in the study can be found in Table 1. It was observed that there were no significant differences in age and BMI between the control group and both the RIF and RPL groups. There showed a significant reduction (*p* < 0.05) in TMPRSS2 expression was observed in the RIF group compared to the control group. On the other hand, the expression of BSG was significantly increased (*p* < 0.05) in the RPL group compared to the control group. No significant differences in the expression of ACE2 and CTSB were found between the groups (*p* > 0.05) (Figures 3 A–H).

**TABLE 1:** Demographics of RIF patients, RPL patients and fertile controls for bioinformatics analysis.

	Fertile controls (n=24)	RIF Patients (n=24)	RPL Patients (n=24)	P 1Value	P 2Value
Age, years	31.1 ± 3.9	32.8 ± 2.3	31.9 ± 3.2	NS	NS
BMI, kg/m2	23.2 ± 1.6	25.4 ± 1.4	23.9 ± 1.34	NS	NS
Parity	1.8 ± 0.5	None	None	<0.05	<0.05

P 1Value: t-test between Control and RIF; P 2Value: t-test between Control and RPL.

Abbreviations: RIF: recurrent implantation failure; RPL: recurrent pregnancy loss; NS: nonsignificant; BMI: body mass index.

### Immunohistochemical analysis of TMPRSS2 and BSG in human endometrium

**FIGURE 4:**
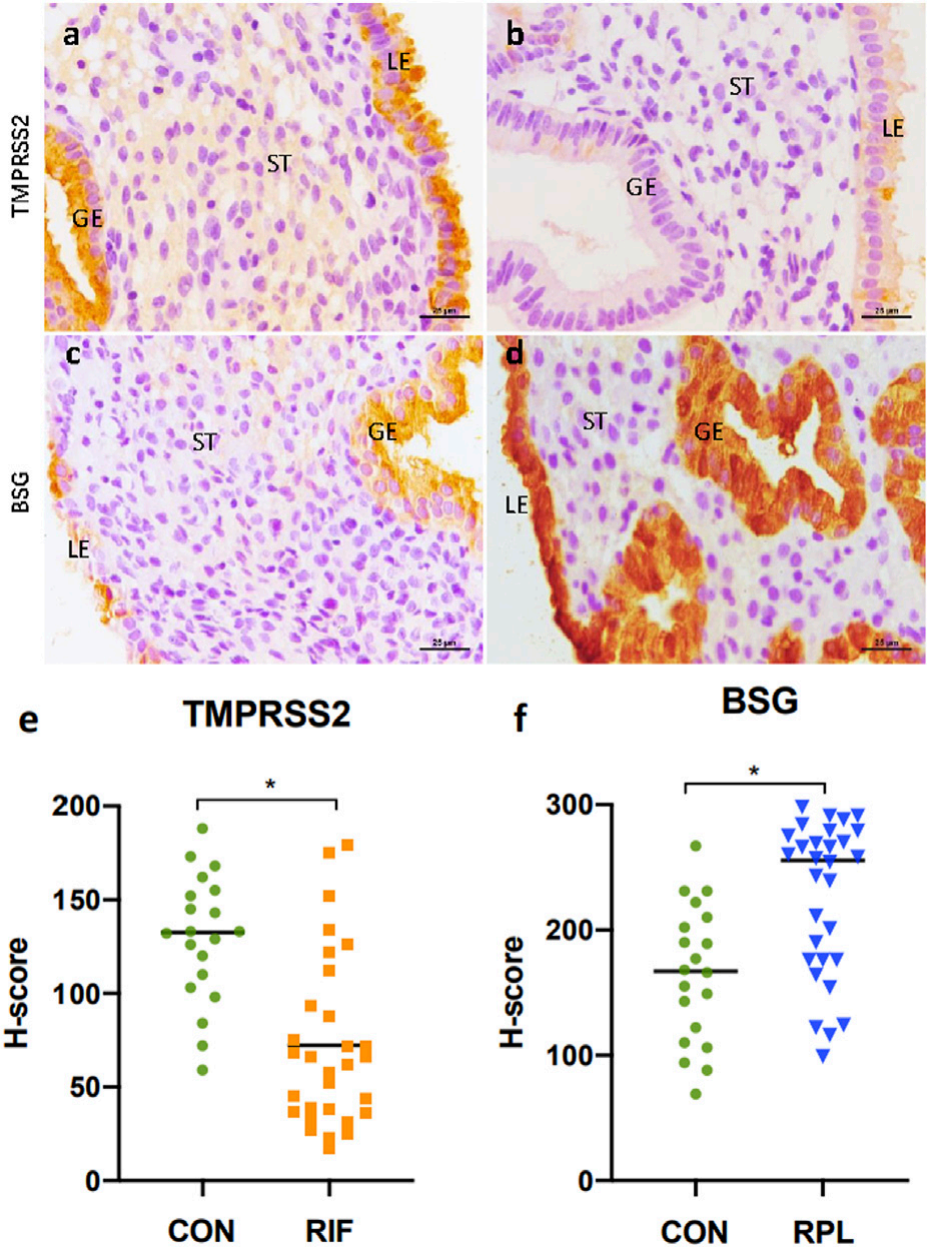
Immunohistochemical staining results of TMPRSS2 in endometrium samples from the control group **(A)** and the RIF group **(B)**, as well as BSG in endometrium samples from the control group **(C)** and the RPL group **(D)**. Both TMPRSS2 and BSG was expressed primarily in the luminal and glandular epithelium. Notably, there was a significant reduction (*p* < 0.05) in TMPRSS2 expression in the RIF group compared to the control group **(E)**, while the expression of BSG was significantly increased (*p* < 0.05) in the RPL group **(F)**.

To validate the expression of TMPRSS2 and BSG in endometrium, immunohistochemical staining was performed on samples obtained from different groups (Figure 4). The detailed demographic characteristics of the women who participated in our study are provided in Table 2. Age and BMI were found to be comparable between control group and both the RIF and RPL groups.

**TABLE 2:** Demographics of RIF patients, RPL patients and fertile controls for IHC.

	Control(n=20)	RIF (n=30)	RPL (n=30)	p value^a^	p value^b^
Age	32.3 ± 2.8	34 ± 3.1	33 ± 3.2	NS	NS 11
BMI(kg/m2)	21.5 ± 2.2	21.7 ± 2.4	22.2 ± 2.5	NS	NS 11
Primipara	12 (60%)	3 (10%)	6 (20%)	<0.01	<0.01
Multipara	8 (40%)	0 (0%)	0 (0%)	<0.01	<0.01
Number of failed cycles					
0	20/20(100%)	0/30 (0%)	30/30(100%)	N/a	N/a
1	0/20 (0%)	0/30 (0%)	0/30 (0%)	N/a	N/a
2	0/20 (0%)	0/30 (0%)	0/30 (0%)	N/a	N/a
3	0/20 (0%)	30/30(100%)	0/30 (0%)	N/a	N/a
No. of previous miscarriages					
0	20/20(100%)	24/30(80%)	0/30 (0%)	N/a	N/a
1	0/20 (0%)	6/30 (20%)	0/30 (0%)	N/a	N/a
2	0/20 (0%)	0/30 (0%)	0/30 (0%)	N/a	N/a
3	0/20 (0%)	0/30 (0%)	30/30(100%)	N/a	N/a

The data are presented as mean±SEM or n/N (%). 11

BMI, body mass index; Control, normal fertile control; RM, recurrent miscarriage; RIF, recurrent implantation failure;

^a^ t-test or Chi-square test to compare between Control and RIF groups ;

^b^ t-test or Chi-square test to compare between Control and RPL groups; N/a, not applicable.

## Discussion

The present study provides valuable insights into the expression patterns of viral infectivity genes in the endometrium, particularly in relation to recurrent implantation failure and recurrent pregnancy loss. Among the viral infectivity genes examined, ACE2, BSG, TMPRSS2, TMPRSS4, CTSB, CTSL, FURIN and MX1 were found in the endometrium. Particularly, CTSB and BSG exhibited the highest expression levels in unciliated epithelium cells, stromal fibroblasts (STF) and smooth muscle cells (SMC). Furthermore, during different menstrual phases, CTSB expression was elevated in the early secretary phase, while BSG expression increased towards the window of implantation, peaking in middle secretary phase. These findings shed light on the dynamic regulation of viral infectivity genes in the endometrium throughout the menstrual cycle. Moreover, our study observed significantly reduced expression of TMPRSS2 in the endometrium of individuals with RIF, while BSG expression was significantly increased in cases of RPL. To validate these findings, immunohistochemistry staining was performed, providing further confirmation.

Vertical transmission of severe acute respiratory syndrome coronavirus 2 during pregnancy is a matter of great interest and concern. Previous studies have reported conflicting evidence regarding vertical transmission. The presence of ACE2 and TMPRSS2 was identified in human trophectoderm of early embryos, extending across all three pregnancy trimesters (Cui et al., 2021). Conversely, ACE2 and TMPRSS2 expression displayed a negative correlation with gestational age, exhibiting elevated levels in trophoblastic tissues of the first trimester and minimal to undetectable levels in third-trimester placenta (Bloise et al., 2021). Therefore, the uterine environments during preconception and early pregnancy could potentially be susceptible to SARS-CoV-2 infection. As a result, the occurrence of COVID-19 in the periconceptional period may heighten the risk of miscarriage. Besides, the interaction between the SARS-CoV-2 virus and ACE2 leads to a downregulation of ACE2, resulting in reduced plasma levels of angiotensin-(1–7). This decrease in angiotensin-(1–7) exacerbates vasoconstriction and promotes a hypercoagulable state. Consequently, these factors contribute to reproductive failures and other complications in obstetrics, such as pre-eclampsia and preterm (Narang et al., 2020; Cook and Ausiello, 2022; Giardini et al., 2022). Furthermore, pregnancy involves immune adaptations to tolerate the developing fetus, while severe cases of COVID-19 exhibit a dysregulated immune response, marked by cytokine storms that lead to an imbalance in Th1/Th2 immune responses (Sills and Wood, 2020; Chang et al., 2021). As a result, the Covid-19-induced “cytokine storm” creates a hypercoagulable condition which adversely affects the typical development of *in utero* blastocysts and fetuses. Moreover, inflammatory markers including C-reactive protein, ferritin, interleukin (IL)-6, IP-10, MCP1, MIP1A, TNF-α, and vWF all were elevated in COVID-19 patients (Zhou et al., 2020). An underlying pro-inflammatory response along with thrombophilic conditions, has been found to be associated with instances of recurrent implantation failure, miscarriage, preeclampsia and preterm birth (Yang et al., 2010; Kwak-Kim et al., 2014; Rana et al., 2019). The incidence of miscarriage was reported to be increased by 25% during the COVID‐19 pandemic (Sacinti et al., 2021). While other studies have found no evidence of vertical transmission of COVID-19 (Chen et al., 2020; Rasmussen et al., 2020). These discrepancies can be attributed to the heterogeneity in disease severity, viral load, duration of COVID-19 positivity, and the presence of other obstetrical risk factors. Understanding the factors influencing fetal outcomes in pregnant individuals infected with SARS-Cov-2 is crucial for optimizing maternal and neonatal care during the ongoing COVID-19 pandemic.

It is well acknowledged that successful implantation process is a result of intricate communications between trophoblastic and decidual cells mediated by multiple cytokines, which form a complex regulatory network to maintain homeostasis between the fetal and the maternal immune systems (Choi and Kwak-Kim, 2008; Saini et al., 2011). The human placental renin-angiotensin system (RAS), directly regulated by ACE2, is reported to be upregulated in the first trimester and involved in endometrial vascularization during the peri-implantation period (Qi et al., 2020). ACE2 and TMPRSS2 have also been recognized in human trophectoderm and placenta at the single-cell level (Cui et al., 2021). Notably, SARS-CoV-2 infection during preconception or the first half of pregnancy may increase the risk of miscarriage by affecting ACE2 activity (Cavalcante et al., 2021). However, previous studies including analysis of gene expression datasets of the endometrium showed no significant expression of ACE2 and TMPRSS2 (Henarejos-Castillo et al., 2020; Cui et al., 2021). Our study similarly found a low percentage (1%) of ACE2 expression in endometrial cells before implantation, with no statistically significant expression difference between fertile women and women with recurrent pregnancy failures. Conversely, TMPRSS2, which cleaves ACE2 at the intracellular C-terminal domain and negatively regulates ACE2 (Heurich et al., 2014; Hoffmann et al., 2020), showed a significant reduction in the endometrium during the peri-implantation period in women with RIF. Nonetheless, it is worth noting that the role of TMPRSS2 in the endometrium may be limited due to its relatively low expression (4%) throughout the menstrual cycle. We also looked into CTSB and BSG, the two most highly expressed SARS-CoV-2-related molecules in the endometrium, and found no statistically significant expression difference between RIF group and fertile women. Overall, the susceptibility of the endometrium to SARS-CoV-2 infection in women with RIF does not appear to be higher than that in fertile women.

Our transcriptomic analysis revealed an accumulated increase in BSG expression in the human endometrium towards the window of implantation (WOI), reaching its zenith during the middle secretary phase, i.e., the peri-implantation period. Previous reports have emphasized the crucial role of BSG as one of the key molecules involved in regulating embryo implantation (Igakura et al., 1998; Lee et al., 2013; Li and Nowak, 2019), and appropriate level of BSG is indispensable in trophoblast–endometrial cell interaction, differentiation and human cytotrophoblasts invasion (Lee et al., 2013). Our analysis demonstrated a statistically significant increase in BSG expression in the RPL group during the implantation period. This elevated expression of BSG expression in the endometrium during the critical period may indicate and increased susceptibility to SARS‐CoV‐2 infection. Such susceptibility could disrupt the delicate homeostasis in the local endometrium thus leading to pregnancy failure or subsequent adverse pregnancy complications. Moreover, BSG has been reported to contribute to the cytokine storm associated with inflammation by producing assorted pro-inflammatory cytokines, for instance interferon-γ (INF-γ), IL-6, monocyte-chemoattractant protein (MCP-1), and tumor necrosis factor-α (TNF-α) (Peng et al., 2020). In the context of COVID, a myriad of complications are not directly caused by the viral infection, but rather result from the dysregulated immune system (Cao, 2020). And the severity of COVID-19 in patients is believed to be associated with an exaggerated immune response and intense inflammation due to the cytokine storm (Wang et al., 2020b; Park, 2021). Another interesting fact should be taken into account is that an unbalanced inflammatory and anti-inflammatory cellular immune response is considered one of the leading causes of miscarriage (Bansal, 2010; Giannubilo et al., 2012). Therefore, the higher expression of BSG in the endometrium may exacerbate the unbalanced inflammation and cytokine storm situation in women with a history of RPL during COVID-19 infection. To summarize, the RPL group are under a higher risk of repetitive miscarriages if infected with COVID-19, either as a consequence of an excessive maternal immune response triggered by elevated endometrial BSG, or simply due to a heightened susceptibility to the virus induced by high levels of BSG in endometrium.

This study possessed several strengths. Firstly, we depicted the expression characteristics of SARS-CoV-2 infectivity-related genes at the single cell level in the endometrium. Secondly, we are the first to investigate the differential expression of SARS-CoV-2 infectivity-related genes in the endometrium of women with RIF and RPL using RNA-seq data. Compared with previous studies (Henarejos-Castillo et al., 2020; Vilella et al., 2021), our study included extended datasets beyond normal endometrium, identified potential biomarkers, validated by other methods and indicated some clinical implications in reproductive failure. Moreover, the differential expressions observed were further confirmed through immunohistochemical staining at the protein level.

However, this study also has certain limitations. The bioinformatic analysis of RIF and RPL was based on a limited number of samples, which necessitates further exploration to determine whether the expression patterns of BSG and TMPRSS2 could be extrapolated to the general population. Additionally, we did not include positive pregnant cases to further demonstrate our findings. Moreover, microarray technology has certain limitations, particularly when it comes to detect genes with low expression levels. But our choice of SARS-CoV-associated factors was guided by the existing literature and their known interactions between these factors and the SARS-CoV-2 virus and significance in viral entry, replication, and pathogenesis.

Despite these limitations, our current study should raise the awareness regarding the potential risks of SARS-CoV-2 infection on women reproduction health. We recommend that affected patients, especially those with a history of RPL, consider postponing *in vitro* fertilization (IVF) treatment or cryopreserving embryo(s) until the mother has recovered from the infection. For pregnant women already in gestation, additional health screenings are suggested, especially for those with a history of RPL during the COVID-19 pandemic.

## Conclusion

Our study revealed a notable upregulation of BSG expression in the endometrium of women experiencing RPL. This finding suggests that this group of women may exhibit an increased susceptivity to SARS-CoV-2 infection within the endometrium, potentially resulting in adverse pregnancy outcomes, such as intrauterine fetal infection or subsequent pregnancy loss. These observations shed light on the complex interplay between BSG expression, viral infectivity, and pregnancy complications, emphasizing the need for further research to elucidate the underlying mechanisms and develop appropriate preventive strategies.

## Data Availability

The datasets presented in this study can be found in online repositories. The names of the repository/repositories and accession number(s) can be found in the article/supplementary material.
